# Modified Gini Index Detector for Cooperative Spectrum Sensing over Line-of-Sight Channels

**DOI:** 10.3390/s23125403

**Published:** 2023-06-07

**Authors:** Dayan Adionel Guimarães

**Affiliations:** National Institute of Telecommunications—Inatel, Av. João de Camargo 510, Santa Rita do Sapucaí 37540-000, MG, Brazil; dayan@inatel.br; Tel.: +55-35-3471-9227

**Keywords:** cognitive radio, dynamic spectrum access, dynamic spectrum sharing, Gini index detector, spectrum sensing

## Abstract

Recently, the Gini index detector (GID) has been proposed as an alternative for data-fusion cooperative spectrum sensing, being mostly suitable for channels with line-of-sight or dominant multi-path components. The GID is quite robust against time-varying noise and signal powers, has the constant false-alarm rate property, can outperform many the state-of-the-art robust detectors, and is one of the simplest detectors developed so far. The modified GID (mGID) is devised in this article. It inherits the attractive attributes of the GID, yet with a computational cost far below the GID. Specifically, the time complexity of the mGID obeys approximately the same run-time growth rate of the GID, but has a constant factor approximately 23.4 times smaller. Equivalently, the mGID takes approximately 4% of the computation time spent to calculate the GID test statistic, which brings a huge reduction in the latency of the spectrum sensing process. Moreover, this latency reduction comes with no performance loss with respect to the GID.

## 1. Introduction

The massive deployment of wireless communication systems in recent years has caused a shortage of the radio-frequency (RF) spectrum. This shortage can be credited to the fixed spectrum allocation policy, in which a primary user (PU) network is given exclusive rights to use a specific RF band. However, research indicates that many allocated RF bands are not fully utilized in certain regions and time periods, leading to inefficient spectrum usage [1,2,3]. As the Internet of Things (IoT) and 5G/6G networks continue to expand, the RF spectrum scarcity issue is expected to worsen, since these technologies require higher bandwidths and will intensify the competition for the limited spectrum resources [4].

One potential solution to the problem of inefficient use of the RF spectrum is the adoption of cognitive radio (CR) networks [5]. These networks are capable of identifying vacant bands resulting from the primary network’s varying spectrum occupation in time and space. A dynamic spectrum access (DSA) policy can be adopted in this case. In DSA, cognitive secondary user (SU) terminals opportunistically make use of unoccupied RF bands. The technique used by the secondary network to utilize vacant bands is called spectrum sensing. This technique is extensively covered in the literature; see for example [3,4,6,7,8] and references therein.

Spectrum sensing can be performed independently by each SU, or can apply multiple SUs in collaboration. When performed by each SU, it can have its performance penalized by propagation-related phenomena, such as multi-path fading, shadowing due to signal blockage, and hidden terminals caused by PU signals not arriving at an SU with detectable strength. On the other hand, cooperative spectrum sensing (CSS) applies several SUs to improve the accuracy of decisions upon the channel occupation state. Thus, CSS has been considered the preferred solution for detecting vacant spectrum bands.

In this paper, a centralized CSS with data fusion is considered, in which the samples of the PU signal received by the SUs are forwarded to the FC, where test statistic computation and comparison with a decision threshold are performed to yield the global decision on the occupation state of the sensed band. This decision is then broadcast to the SUs, which subsequently compete for the band if it is vacant using an appropriate multiple-access technique.

### 1.1. Related Research

The literature is rich in research works proposing detectors for spectrum sensing. Many of them apply the sample covariance matrix (SCM) as the received PU signal, as described for instance in [9,10,11,12,13,14,15] and references therein. These detectors are usually referred to as covariance-based detectors.

Blind detectors are considered herein. They do not make use of any information about the detected signal or the noise variance. Examples are the Hadamard ratio (HR) detector [9], the arithmetic to geometric mean (AGM) detector [10], volume-based detectors (VD), number 1 (VD1) and number 2 (VD2) [11], the maximum–minimum eigenvalue detector (MMED) [12], the eigenvalue-based generalized likelihood ratio test (GLRT) [12] detector, the Gini index detector (GID) [13], the Gerschgorin radii and centers ratio (GRCR) [14] detector, and the Pietra–Ricci index detector (PRIDe) [15].

The computational burdens associated with the calculation of the test statistics of the above-mentioned detectors differ, depending on the operations needed. For example, the HR and VD are based on the determinant of the SCM. The AGM, the MMED, and the GLRT need the estimation of the eigenvalues of the SCM. On the other hand, the GID, GRCR, and PRIDe operate directly on the elements of the SCM, and as a consequence are less complex.

The GID, GRCR, and PRIDe are particularly noteworthy, not only because they have lower computational complexity, but also because they are more robust against signal and noise power variations than the other detectors considered herein.

The GID detector is further explored in this work. Its test statistic is based on the Gini index, which is a statistical dispersion metric commonly used in economics and social sciences as a measure of inequality [13]. Although the GID test statistic does not make use of eigenvalues or determinants of the SCM, its computational cost is penalized due to the relatively high burden associated with the calculation of the magnitude of the elements of the SCM, or the magnitude of the results of operations performed on the SCM elements. This work aims at reducing the computational cost of the GID detector, which is achieved by replacing with real quantities the complex values operated in the test statistic while maintaining the original Gini-index-based operations defined in [13].

### 1.2. Contribution and Structure of the Article

The modified Gini index detector (mGID) is proposed in this paper. Likewise, the GID, the test statistic of the mGID, is formed from the elements of the SCM, which makes it less complex than most of the state-of-the-art detectors proposed so far. The mGID inherits important attributes of the GID, namely, blindness, attractive robustness against nonuniform and time-varying received signal and noise levels, and a constant false-alarm rate (CFAR). Moreover, its performance is practically equal to the GID’s. In summary, the main contributions of the present work are as follows:

- The novel and practical mGID test statistic, whose computational cost is drastically reduced when compared to the GID, with practically no performance loss and inherited desirable attributes of the GID.
- A two-fold model improvement with respect to [13] time-varying signal power attached to path-loss and corrected signal-to-noise ratio (SNR) calibration.
- Numerical results that provide insight into the system’s operation and support the conclusions of the work.
- A fully-commented Matlab simulation code [16] capable of supporting a complete system analysis under a variety of circumstances and system parameters.

The remainder of the article is organized as follows: Section 2 describes the signal, noise, and channel models. The GID and mGID test statistics are addressed in Section 3. Numerical results and interpretations are presented in Section 4. The conclusions and opportunities for future research are given in Section 5.

## 2. Signal, Noise, and Channel Models

The model for centralized CSS with data fusion adopted herein is grounded on [13,15]. It considers *m* SUs in cooperation, each one collecting *n* samples of the PU signal during a sensing interval. The samples gathered by the SUs are transmitted to the FC over an error-free report channel, forming the sample matrix $Y\in C^{m\times n}$, which is given by

(1) $$Y=hx^{T}+V,$$

where the vector $x\in C^{n\times 1}$ contains the PU signal samples, which can be modeled as zero-mean complex Gaussian random variables or modulated signal samples in the base band. Here, a base-band quaternary phase-shift keying (QPSK) PU signal is adopted.

The channel vector $h\in C^{m\times 1}$ in (1) is formed by elements, $h_{i}$, that represent the sensing channel gains between the PU transmitter and the *i*th SU, for i=1,…,m. The variation in these gains over time can model the fading effect produced in the signal due to multi-path propagation of the electromagnetic wave and other propagation-related variations imposed on the PU signal, such as shadowing due to blockage by obstacles.

When modeling spectrum sensing, the gains of the channels between the PU transmitter and the SU’s receivers are often considered constant during the sensing interval, and independent and identically distributed among consecutive sensing events. The constant channel gains mean that the duration of the sensing interval is shorter than the coherence time [17] of the sensing channel, that is, the signal fading produced by the channel changes slowly and can be considered practically constant during the entire sensing interval. On the other hand, the independence between successive sensing events means that the interval between these events is greater than the coherence time of the sensing channel. Additionally, the multiplication between h and $x^{T}$ in (1) is meant to model a flat fading channel whose coherence bandwidth is larger than the bandwidth of the PU signal [17].

The channel vector is modeled as h=Ga, where G is a gain matrix to be defined a little later, and $a\in C^{m\times 1}$ is a vector formed by complex-Gaussian random variables $a_{i}∼CN\left[\sqrt{K/(2K+2)},1/\left(K+1\right)\right]$, where $K=10^{K^{\left(\mathrm{dB}\right)}/10}$ is the Rice factor [17] of the channels between the PU transmitter and the SUs, and where $K^{\left(\mathrm{dB}\right)}=10\log_{10}\left(K\right)$ is the Rice factor in decibels.

When a channel is subjected to Ricean fading, the Rice factor is a measure of the ratio between the signal strength received via the dominant propagation path and the power of the signals coming from the other paths. A dominant propagation path occurs, for example, in a line-of-sight (LoS) condition between the transmit and receive antennas, or when a strong specular reflection of the transmitted signal towards the receiver happens. A larger Rice factor is associated with less variability (i.e., less fading) in the instantaneous received signal strength. A zero Rice factor ($K^{\left(\mathrm{dB}\right)}=-\infty$ dB) corresponds to a channel with Rayleigh fading. On the other hand, a pure additive white Gaussian noise (AWGN) channel results if $K^{\left(\mathrm{dB}\right)}=\infty$ dB. In practical terms, $K^{\left(\mathrm{dB}\right)}<-10$ dB corresponds to an approximate Rayleigh fading, and $K^{\left(\mathrm{dB}\right)}>10$ dB makes the channel approximately free of fading.

From measurements reported in [18], it has been found that $K^{\left(\mathrm{dB}\right)}$ is an environment-dependent random variable that can be well characterized by a Gaussian distribution with mean $\mu_{K}$ and standard deviation $\sigma_{K}$, both in dB, i.e., $K^{\left(\mathrm{dB}\right)}∼N\left[\mu_{K},\sigma_{K}\right]$. Typical values of $\mu_{K}$ and $\sigma_{K}$ are determined according to the propagation characteristics of the area, and are reported in [18] as well. This model for the Ricean fading is consistent with reality: While moving, an SU receiver experiences different situations in terms of the line of sight with the PU transmitter antenna. For example, urban areas typically have a large number of big obstacles between the PU and the SUs, which reduces the mean Rice factor and increases its variability, which is measured by $\sigma_{K}$. On the other hand, rural or open areas exhibit higher Rice factors, on average, but have lower variability due to the smaller number and small diversity of the obstacles commonly found in these areas. An urban area typically has $\mu_{K}=1.88$ dB and $\sigma_{K}=4.13$ dB. Rural or open areas typically have $\mu_{K}=2.63$ dB and $\sigma_{K}=3.82$ dB. Suburban regions are commonly associated with $\mu_{K}=2.41$ dB and $\sigma_{K}=3.84$ dB [18].

The present signal model also takes into account that the received signal power levels across the SUs may be unequal and time-varying due to the different distances between the PU transmitter and the SUs, and due to the variation in theses distances across different sensing events caused by the motion of the SUs. To model unequal and time-varying received signal levels, the above-mentioned gain matrix $G\in R^{m\times m}$ is given by $G=\mathrm{diag}(\sqrt{p/P_{\mathrm{tx}}})$, where $p={\left[{P_{\mathrm{rx}}}_{1},\cdots ,{P_{\mathrm{rx}}}_{m}\right]}^{T}$ is the vector containing the received PU signal powers across the *m* SUs, with ${\left[\cdot \right]}^{T}$ denoting transposition, $P_{\mathrm{tx}}$ is the transmitted PU signal power in watts, and the operator diag(·) returns a diagonal matrix whose diagonal is formed by the elements of the vector in the argument.

The log-distance path-loss prediction model [17] represents a very simple form to calculate the area-mean received signal power at a distance *d* from the transmitter. It can be used to calculate the PU signal power received by the *i*th SU, ${P_{\mathrm{rx}}}_{i}$, yielding

(2) $${P_{\mathrm{rx}}}_{i}=P_{\mathrm{tx}}{\left(\frac{d_{0}}{d_{i}}\right)}^{\eta },$$

where $d_{0}$ is a reference distance in the far-field region of the PU transmit antenna, $d_{i}$ is the distance between the PU transmitter and the *i*th SU receiver, and η is the environment-dependent dimensionless path-loss exponent [17]. The larger the value of η, the grater the signal attenuation at a given distance.

Variability in thermal noise at the SUs’ receivers is also assumed in the present model. In practice, this variability cannot be neglected and is caused by multiple factors, such as variations in the ambient temperatures to which the SUs are subjected, uncalibrated receiver front-end circuits, uneven noise figures and gains of the low noise amplifiers (LNAs), and unwanted signals present in the sensed band, such as atmospheric noise and RF interference, which can be considered as background noise added to the receiver thermal noise. In this paper, the nonuniform and time-varying characteristic of the noise power is modeled as follows: since the elements in the *i*th row of the matrix $V\in C^{m\times n}$ in (1) are associated with the *i*th SU, they are represented by independent Gaussian random variables whose mean is zero and the variance is

(3) $$\sigma_{i}^{2}=\left(1+\rho u_{i}\right){\bar{\sigma }}^{2},$$

where $u_{i}$ is a realization of a uniform random variable $U_{i}$ in the interval [−1,1], that is, $U_{i}∼U\left[-1,1\right]$, ${\bar{\sigma }}^{2}$ is the noise variance averaged across all SUs, and 0≤ρ<1, which is a predefined parameter of the model, is the fractional variation in the noise power around ${\bar{\sigma }}^{2}$.

The instantaneous signal-to-noise ratio, γ, across the SUs is a random variable, since it depends on $\sigma_{i}^{2}$ and on the distances $d_{i}$, which vary according to the random SUs’ locations. In light of (2) and (3), a realization of γ is given by

(4) $$\gamma =\frac{1}{m}\sum_{i=1}^{m}\frac{P_{\mathrm{tx}}{\left(d_{0}/d_{i}\right)}^{\eta }}{\left(1+\rho u_{i}\right){\bar{\sigma }}^{2}}.$$

Thus, the average SNR across the SUs, which is also a predefined parameter of the model, is given by

(5) SNR=E[γ],

where E[γ] is the expected value of γ.

To implement this variable noise level model, firstly the expectation of the uncalibrated $\gamma^{\prime }$ has to be calculated, which is defined for an uncalibrated average noise variance ${\bar{\sigma }}^{2}=1$ and for a given realization of the SUs’ locations. Taking into account that the random variables $U_{i}∼U\left[-1,1\right]$ are independent from each other, it can be written, in light of (4), that

(6) $$E\left[\gamma^{\prime }\right]=\frac{1}{m}\sum_{i=1}^{m}E\left[\frac{{P_{\mathrm{rx}}}_{i}}{\left(1+\rho U_{i}\right)}\right],$$

where the uniform random variable $Z_{i}=1+\rho U_{i}$ lies in-between a=1−ρ and b=1+ρ.

The expectation of the random variable ${P_{\mathrm{rx}}}_{i}/Z_{i}$ is given by

(7) $$E\left[\frac{{P_{\mathrm{rx}}}_{i}}{Z_{i}}\right]=\int_{a}^{b}\frac{{P_{\mathrm{rx}}}_{i}}{z_{i}\left(b-a\right)}dz_{i}=\frac{{P_{\mathrm{rx}}}_{i}}{b-a}\ln\left(\frac{b}{a}\right).$$

Applying this result in (6), with a=1−ρ and b=1+ρ, the expected value of $\gamma^{\prime }$ for 0<ρ<1 is found to be

(8) $$E\left[\gamma^{\prime }\right]=\frac{\ln\left(\frac{1+\rho }{1-\rho }\right)}{2\rho m}\sum_{i=1}^{m}{P_{\mathrm{rx}}}_{i}.$$

For ρ=0, Equation (8) yields an indeterminate result that can be easily solved taking into account that

$$\lim_{\rho \to 0}\frac{1}{2\rho }\ln\left(\frac{1+\rho }{1-\rho }\right)=1,$$

in this case yielding

(9) $$E\left[\gamma^{\prime }\right]=\frac{1}{m}\sum_{i=1}^{m}{P_{\mathrm{rx}}}_{i}.$$

Since $\mathrm{SNR}=E\left[\gamma \right]=E\left[\gamma^{\prime }\right]/{\bar{\sigma }}^{2}$, the calibrated average noise variance is given by

(10) $${\bar{\sigma }}^{2}=\frac{E[\gamma^{\prime }]}{\mathrm{SNR}}.$$

In the last step, this ${\bar{\sigma }}^{2}$ is plugged into (3), along with a realization $u_{i}$ of the random variable $U_{i}$, to yield $\sigma_{i}^{2}$, which is the variance in the noise samples in the *i*th row of the matrix V. New values of $\left\{\sigma_{i}^{2}\right\}$ are computed in each sensing round, conferring the time-varying character to the noise power.

**Figure 1:**
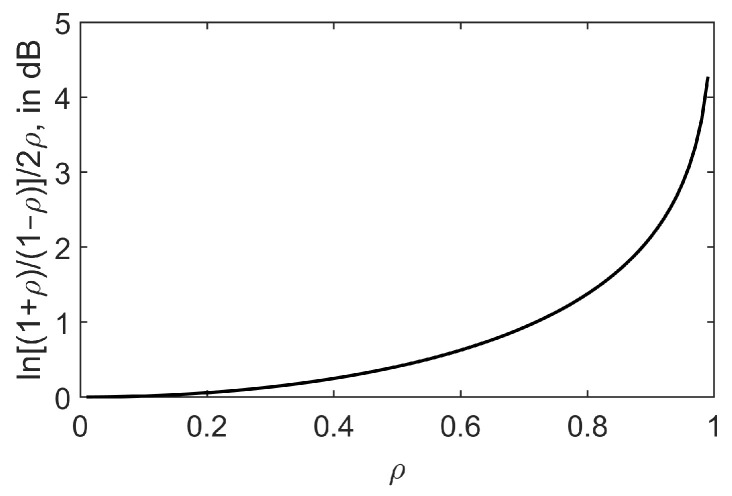
Factor ln[(1+ρ)/(1−ρ)]/2ρ, in dB, as a function of ρ.

The present SNR calibration model corrects the one considered in [13], in which the SNR is given by the ratio between the average signal power and the average noise power, which could be better named average signal to average noise power ratio. Notice from (4) and (5) that the correct SNR calculation is the average of the quotient between the instantaneous signal power and the instantaneous noise power. Using the notation adopted herein, in [13] it is considered that

$$\mathrm{SNR}=\frac{1}{m}\sum_{i=1}^{m}\frac{P_{{\mathrm{rx}}_{i}}}{E[\left(1+\rho U_{i}\right){\bar{\sigma }}^{2}]}=\frac{1}{m{\bar{\sigma }}^{2}}\sum_{i=1}^{m}P_{{\mathrm{rx}}_{i}},$$

and here, it is established that

$$\mathrm{SNR}=\frac{1}{m}\sum_{i=1}^{m}E\left[\frac{P_{{\mathrm{rx}}_{i}}}{\left(1+\rho U_{i}\right){\bar{\sigma }}^{2}}\right]=\frac{\ln\left(\frac{1+\rho }{1-\rho }\right)}{2\rho m{\bar{\sigma }}^{2}}\sum_{i=1}^{m}{P_{\mathrm{rx}}}_{i},$$

where the factor ln[(1+ρ)/(1−ρ)]/2ρ makes explicit the inequality between the two SNR definitions. This factor is plotted in Figure 1, in dB, as a function of ρ. If the SNR is defined according to [13] so that it remains constant for any ρ, the actual SNR will be $10\log_{10}\left\{\ln\left[\left(1+\rho \right)/\left(1-\rho \right)\right]/2\rho \right\}$ dB higher, progressively overestimating the detector’s performance in regard to the one achieved with the correct SNR, as ρ becomes larger.

The influence of the correct SNR calibration on the detector’s performance is evidenced by means of a numerical result presented in Section 4, specifically by Figure 7.

## 3. Test Statistics, Time Complexities, and Constant False-Alarm Rate of GID and mGID

This section presents the GID test statistic and the derivation of its modified version, the mGID test statistic. A time complexity analysis is subsequently performed by comparing the computer run time of both detectors. Finally, the constant false-alarm rate properties of the GID and mGID are addressed.

### 3.1. GID and mGID Test Statistics

The matrix Y defined in (1), which contains the mn samples received by the *m* SUs, is formed at the FC. Under the hypothesis $H_{0}$, the primary signal is absent in the sensed band, yielding Y=V. Under the hypothesis $H_{1}$, then $Y=hx^{T}+V$. Given Y, the SCM of order *m* is subsequently computed at the FC, according to

(11) $$R=\frac{1}{n}YY^{†},$$

where † denotes complex conjugate and transpose.

Let $r_{i}$ denote the *i*th element of the vector r formed by stacking all columns of R, for $i=1,\ldots ,m^{2}$. The GID test statistic proposed in [13] is computed at the FC according to

(12) $$T_{\mathrm{GID}}=\frac{2\left(m^{2}-m\right)\sum_{i=1}^{m^{2}}\left|r_{i}\right|}{\sum_{i=1}^{m^{2}}\sum_{i=1}^{m^{2}}\left|r_{i}-r_{j}\right|},$$

where the constant $2(m^{2}-m)$ has been used in [13] for convenience, but it does not influence the performance of the GID detector, and can be removed.

The decision on the occupation state of the sensed band is made at the FC by comparing $T_{\mathrm{GID}}$ with a decision threshold λ. If $T_{\mathrm{GID}}>\lambda$, the decision is made in favor of the hypothesis $H_{1}$. Otherwise, $H_{0}$ is chosen. The value of λ is defined a priori, according to the desired false-alarm rate.

The calculation of the magnitude of the complex quantities contained in r is responsible for the main computational burden associated with the test statistic (12). The magnitude calculation carries a relatively high computational cost due to its computation as the square root of the sum of the squared-real and squared-imaginary parts. To aggravate this computational burden, such calculation must be performed $m^{2}$ times in the numerator and $m^{4}$ times in the denominator of (12).

The mGID test statistic proposed in this paper is formed by replacing the possibly complex quantities, $r_{i}$, in (12) by the real quantities $q_{i}=ℜ\left(r_{i}\right)+ℑ\left(r_{i}\right)$, where $ℜ(r_{i})$ and $ℑ(r_{ik})$ are the real and the imaginary parts of $r_{i}$, respectively. As a consequence, the computational cost of the mGID is drastically reduced in comparison with the GID. It is noteworthy that a similar procedure has been used in [19] to devise the modified Pietra–Ricci index detector (mPRIDe). The resultant mGID test statistic is

(13) $$T_{\mathrm{mGID}}=\frac{\sum_{i=1}^{m^{2}}\left|q_{i}\right|}{\sum_{i=1}^{m^{2}}\sum_{j=i}^{m^{2}}\left|q_{i}-q_{j}\right|},$$

from which the constant $2(m^{2}-m)$ has been removed, and an additional simplification has been performed in the right-hand summation of the denominator, which sums from j=i to $j=m^{2}$ instead of the original sum from j=1 to $j=m^{2}$. This simplification takes into account that $|q_{i}-q_{j}\left|=\right|q_{j}-q_{i}|$.

**Figure 2:**
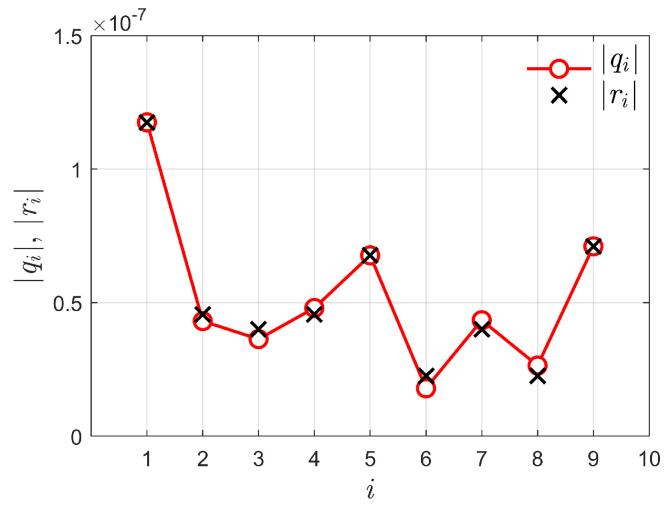
Random realizations of $|r_{i}|$ and $|q_{i}|$ for m=3.

To illustrate the implication of using $|q_{i}|$ instead of $|r_{i}|$, Figure 2 depicts random realizations of $|r_{i}|$ and $|q_{i}|$ for m=3, corresponding to the sample covariance matrix

$$\begin{array}{c} R=\left[\begin{array}{ccc} r_{1} & r_{4} & r_{7}\\ r_{2} & r_{5} & r_{8}\\ r_{3} & r_{6} & r_{9} \end{array}\right]\\ \approx 10^{-6}\left[\begin{array}{ccc} 0.118 & 0.046+j0.003 & 0.040+j0.004\\ 0.046-j0.003 & 0.068 & 0.022+j0.004\\ 0.040-j0.004 & 0.022-j0.004 & 0.071 \end{array}\right]. \end{array}$$

From this figure, it can be seen that the values of $|r_{i}|$ and $|q_{i}|=|ℜ\left(r_{i}\right)+ℑ\left(r_{i}\right)|$ are not pairwise equal, except for the diagonal elements of R, which correspond to $r_{1}=q_{1}$, $r_{5}=q_{5}$, and $r_{9}=q_{9}$. Nonetheless, there is a compensation effect in the sums $\sum_{i}\left|q_{i}\right|$ that can be observed in the values of $\left(q_{2},q_{4}\right)$, $\left(q_{3},q_{7}\right)$, and $\left(q_{6},q_{8}\right)$: notice that $q_{2}$ is shifted below $r_{2}$ approximately the same amount that $q_{4}$ is shifted above $r_{4}$; analogously, $q_{3}$ is shifted below $r_{3}$ approximately the same amount that $q_{7}$ is shifted above $r_{7}$; and $q_{6}$ is shifted below $r_{6}$ approximately the same amount that $q_{8}$ is shifted above $r_{8}$. As a consequence, $\sum_{i}|q_{i}|\approx \sum_{i}\left|r_{i}\right|$, which produces a negligible modification in the numerator of $T_{\mathrm{mGID}}$ with respect to the numerator of $T_{\mathrm{GID}}$. The same reasoning applies to the denominators, that is, $\sum_{i}\sum_{j}|q_{i}-q_{j}|\approx \sum_{i}\sum_{j}\left|r_{i}-r_{j}\right|$. Thus, there is a small difference between $T_{\mathrm{GID}}$ and $T_{\mathrm{mGID}}$, which guarantees that their performances are approximately the same, to be confirmed by the results presented in Section 4.

### 3.2. Time Complexities

Regardless of the magnitude of a real number, its absolute value can be calculated in constant time. Taking this fact into account, the time complexity of (13), using the big-O notation, is $O(m^{4})$, as determined by the computation of its denominator. Analogously, the time complexity of (12) is also $O(m^{4})$. However, (12) is computed with a larger constant factor than in the case of (13) because the absolute value of a complex quantity applies a square-root operation, which is way more computationally expensive than multiplications or additions. Hence, the detectors GID and mGID have the same time complexity in terms of the big-O notation, but the GID test statistic takes longer to be computed due to the higher computational cost of the square-root operation.

**Figure 3:**
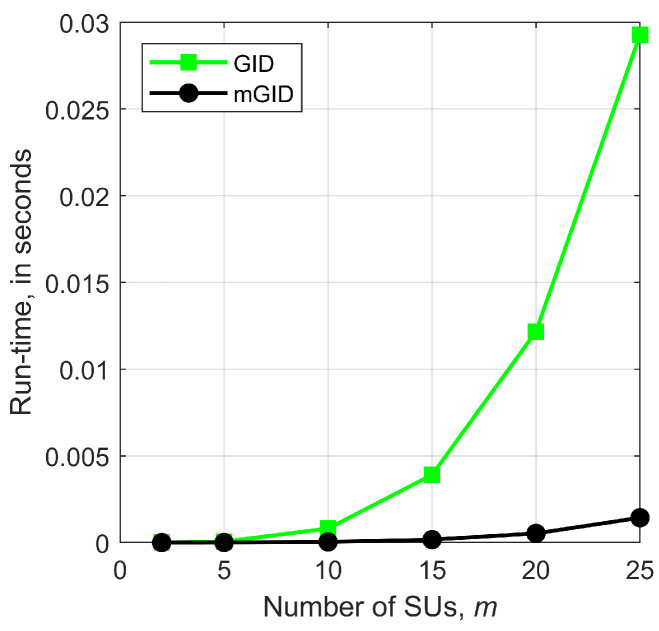
Run-time computation measurements of the GID and mGID test statistics.

In order to assess the time complexities of the GID and mGID test statistics, run-time measurements were carried out for them, as a function of the number of SUs, *m*, using a computer with an 11th Gen Intel Core i7-11800H @ 2.30 GHz processor, with 64 GB RAM, running the Windows 11 Professional and the 64-bit Matlab R2018b, and measured via the Matlab functions tic and toc. These functions were respectively placed before and after the subroutine that computes the corresponding test statistics in the Matlab code [16]. The resultant measurements are shown in Figure 3.

A power curve fitting of the type $ax^{b}+c$ was applied to the run-time measurement results, using the pwrfit function of the Mathcad software [20], version 15, yielding $a_{1}\approx 2.2\times 10^{-7}$, $b_{1}\approx 3.90$, and $c_{1}\approx 3.5\times 10^{-5}$ for the GID, and $a_{2}\approx 9.4\times 10^{-9}$, $b_{2}\approx 3.86$, and $c_{2}\approx 9.3\times 10^{-6}$ for the mGID. From this procedure, it is demonstrated the fourth-power growth rate of the computation time for both detectors, as given by $b_{1}$ and $b_{2}$, approximately equals 4, and the much larger constant time factor of the GID is approximately 23.4 times larger than in the case of the mGID, as given by $a_{1}/a_{2}\approx 23.4$. In other words, the mGID test statistic (13) takes approximately 4% of the computation time spent for calculating the GID test statistic (12), which represents a huge reduction in the latency of the spectrum sensing process.

### 3.3. Constant False-Alarm Rate

The CFAR property is the ability of a detector to maintain the false-alarm rate irrespective the value of a system parameter of interest [19]. In the context of spectrum sensing, the parameter of interest is the noise variance.

**Figure 4:**
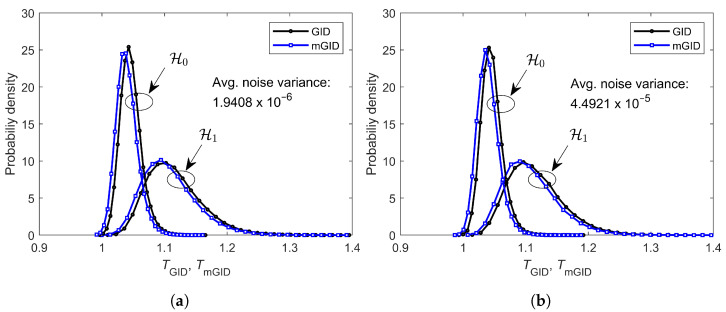
Empirical PDFs of the test statistics $T_{\mathrm{GID}}$ and $T_{\mathrm{mGID}}$ under $H_{0}$ and $H_{1}$: (**a**) average noise variance ${\bar{\sigma }}^{2}=1.9408\times 10^{-6}$; (**b**) average noise variance ${\bar{\sigma }}^{2}=4.4921\times 10^{-5}$.

To assess the CFAR property of the GID and mGID, Figure 4 depicts the empirical probability density functions (PDFs) of $T_{\mathrm{GID}}$ and $T_{\mathrm{mGID}}$, under $H_{0}$ and $H_{1}$, for m=6, SNR=−13 dB, η=2.5, n=250, ρ=0.5, $\mu_{K}=20$ dB, and $\sigma_{K}=0$ dB, for 50,000 realizations of each test statistic, under each hypothesis, and for two different values of the average noise variance ${\bar{\sigma }}^{2}$. The specific noise variances ${\bar{\sigma }}^{2}=1.9408\times 10^{-6}$ and ${\bar{\sigma }}^{2}=4.4921\times 10^{-5}$ resulted from setting the PU transmitted signal power to 5 and 50 watts, respectively.

From Figure 4, it can be seen that the supports of the PDFs of $T_{\mathrm{GID}}$ under $H_{0}$ do not change when subjected to different noise variances, the same occurring with $T_{\mathrm{mGID}}$. Hence, given a predefined decision threshold, the probability of a false alarm will not change with the noise variance, which confers the CFAR property to the GID and mGID.

Figure 4 also suggests that the performances of the GID and mGID are the same, since the PDFs of their test statistics are practically identical in shape, except for a small shift of the mGID’s PDFs to the left, relative to the GID’s.

## 4. Numerical Results

This section addresses the performance of the detectors GID and mGID, as well as of the competing state-of-the-art blind detectors listed in Section 1, namely, PRIDe, HR, VD1 (VD number 1), GLRT, MMED, AGM, and GRCR. The test statistics of these competing detectors are given in the sequel.

The test statistic of the PRIDe detector [15] is

(14) $$T_{\mathrm{PRIDe}}=\frac{\sum_{i=1}^{m^{2}}|r_{i}|}{\sum_{i=1}^{m^{2}}|r_{i}-\bar{r}|},$$

where $r_{i}$ is the same variable defined in (12), and $\bar{r}=\left(1/m^{2}\right)\sum_{i=1}^{m^{2}}r_{i}$.

The HR test statistic [9] is computed as

(15) $$T_{\mathrm{HR}}=\frac{\det(R)}{\prod_{i=1}^{m}r_{i,i}},$$

where $r_{i,j}$ is the element in the *i*-th row and *j*-th column of the sample covariance matrix R, and det(R) is the determinant of R.

The VD1 detector [11] has a test statistic given by

(16) $$T_{\mathrm{VD}1}=\log\left[\det(E^{-1}R)\right],$$

where E=diag(e), with diag(e) being the diagonal matrix whose main diagonal corresponds to the vector $e=[e_{1},e_{2},\cdots ,e_{m}]$, where $e_{i}={∥R\left(i,:\right)∥}_{2}$ and ${∥\cdot ∥}_{2}$ denotes the Euclidean norm.

The test statistic of the eigenvalue-based GLRT detector [12] is

(17) $$T_{\mathrm{GLRT}}=\frac{\lambda_{1}}{\sum_{i=1}^{m}\lambda_{i}},$$

where $\lambda_{1}\geq \lambda_{2}\geq \cdots \geq \lambda_{m}$ are the eigenvalues of R.

The detector MMED [12] forms its test statistic according to

(18) $$T_{\mathrm{MMED}}=\frac{\lambda_{1}}{\lambda_{m}}.$$

In the case of the AGM [10], the test statistic is given by

(19) $$T_{\mathrm{AGM}}=\frac{\frac{1}{m}\sum_{i=1}^{m}\lambda_{i}}{{\left(\prod_{i=1}^{m}\lambda_{i}\right)}^{1/m}},$$

and the GRCR [14] has a test statistic computed according to

(20) $$T_{\mathrm{GRCR}}=\frac{\sum_{i=1}^{m}\sum_{j=1,j\neq i}^{m}\left|r_{i,j}\right|}{\sum_{i=1}^{m}r_{i,i}}.$$

The performances reported herein have been measured in terms of the probability of detection, $P_{d}$, for a fixed $P_{\mathrm{fa}}=0.1$, as a function of the main system parameters. All results were generated via Monte Carlo computer simulations using the Matlab R2018a running the source code available in [16].

**Figure 5:**
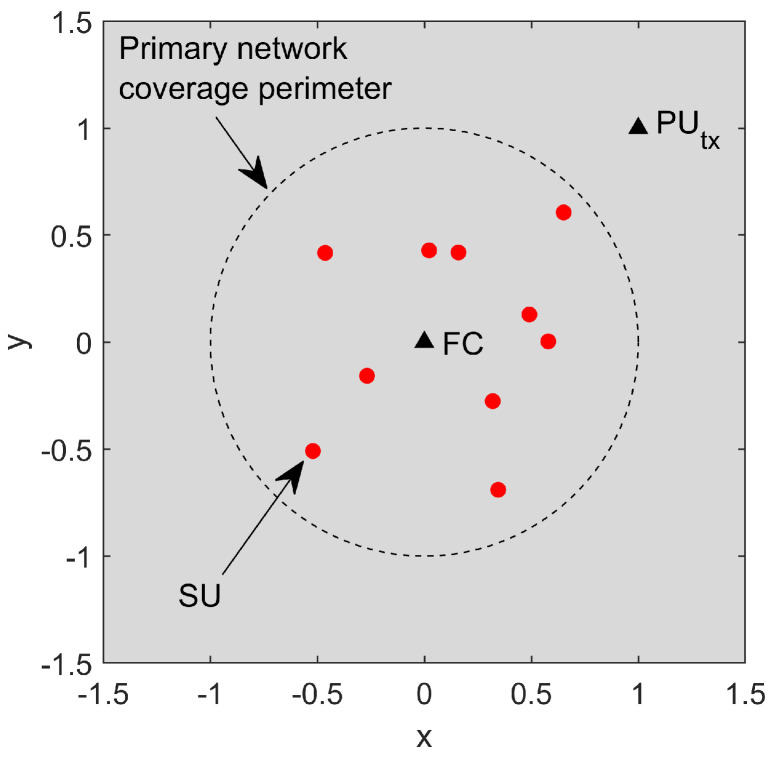
CSS topology for m=10 SUs, normalized coverage radius r=1 m, FC at (x,y)=(0,0) m, and PU transmitter at (x,y)=(1,1) m.

The CSS topology adopted for analysis is exemplified in Figure 5. It comprises a secondary network with *m* SUs (m=10 in this example) uniformly distributed in a circular coverage area with radius *r* meters (a normalized r=1 has been used in this figure), with the PU transmitter located at (x,y)=(r,r) meters, with (x,y)=(1,1) meter in the figure, and the FC located at the center of the coverage area. Each sensing event requires defining new random positions of the SUs, aiming at mimicking mobile SUs. The PU signal samples, which come from a base-band QPSK signal, are then generated according to the model described in Section 2 for the vector x. These samples are multiplied by the channel vector h and the result is added to the noise matrix V, according to (1), forming the matrix Y of received signal samples. As demonstrated in Section 2, the channel vector h accounts for multi-path fading and distance-dependent signal attenuation from the PU transmitter to the SUs. Subsequently, matrix Y is made available to the FC, where the sample covariance matrix R is computed, and from which the desired test statistics are formed. For each detector under analysis, a number of 20,000 test statistic values are computed under $H_{0}$ and $H_{1}$, and used to estimate the cumulative distribution functions (CDFs) from which $P_{d}$ is read for the reference $P_{\mathrm{fa}}=0.1$.

**Figure 6:**
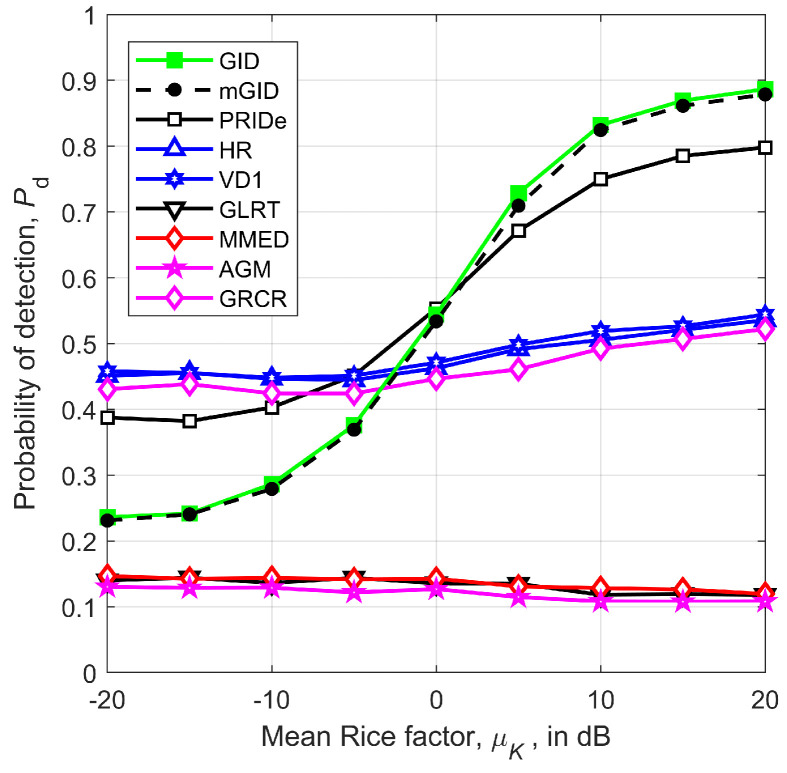
$P_{d}$ versus $\mu_{K}$, for m=6, SNR=−13 dB, η=2.5, r=1 km, n=250, ρ=0.5, and $\sigma_{K}=0$ dB.

Figure 6 depicts $P_{d}$ versus the mean of the Rice factor of the sensing channel, $\mu_{K}$, for m=6 SUs, SNR=−13 dB, path-loss exponent η=2.5, coverage radius r=1 km, n=250 samples per SU, fraction of noise power variation ρ=0.5, and standard deviation of the Rice factor $\sigma_{K}=0$ dB.

The main result unveiled by Figure 6 is the influence of the line-of-sight condition from the PU transmitter to the SUs on the performance of the detectors, especially in the case of the GID and mGID. Firstly, notice that the GID and mGID achieve practically the same performances. Moreover, they become attractive under a moderate-to-high LoS component, i.e., for $\mu_{K}>0$ dB. Taking this fact into consideration, the remaining results of this section consider $\mu_{K}=20$ dB, which tightly approximates a pure AWGN channel. This is to explore the GID and mGID operations under the scenario corresponding to their best performances.

Figure 6 also demonstrates that the performance variation pattern of the PRIDe is similar to the GID and mGID, yielding a better performance for lower values of $\mu_{K}$ and a worse performance for higher values of $\mu_{K}$. The other detectors exhibit low sensitivity to the variation in $\mu_{K}$, with the GLRT, MMED, and AGM being practically useless. This is because these three detectors are not robust against variations in the received signal and noise levels, which are implicitly present in the setup used to plot Figure 6.

**Figure 7:**
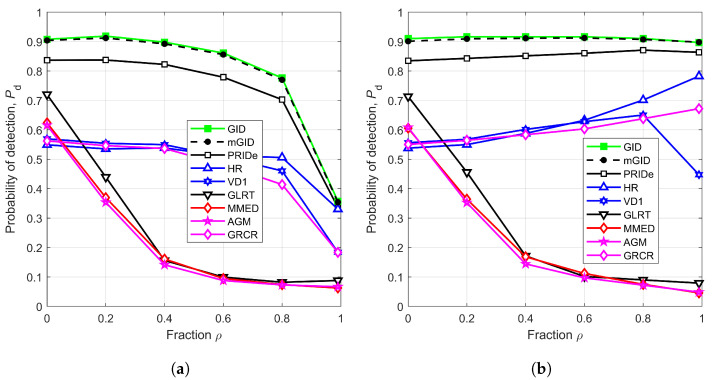
$P_{d}$ versus ρ, for m=6, SNR=−13 dB, η=2.5, r=1 km, n=250, $\mu_{K}=20$ dB, and $\sigma_{K}=0$ dB: (**a**) correct SNR model; (**b**) incorrect SNR model.

The effect on $P_{d}$ due to changes in the fraction ρ that governs the amount of noise power variation about its average is shown in Figure 7, for m=6, SNR=−13 dB, η=2.5, r=1 km, n=250, $\mu_{K}=20$ dB, and $\sigma_{K}=0$ dB. Figure 7a considers the new model for calibrating the SNR, according to (5) and related equations, while Figure 7b has been plotted under the SNR definition given in [13], which corresponds to the ratio between the average signal power to the average noise power. Notice that the performances of the GID and mGID are practically the same, and that they are quite robust against the variation in the noise level, up to ρ=0.8, the same occurring with the detectors PRIDe, HR, VD1, and GRCR. The performance of the detectors GLRT, MMED, and AGM are highly affected by the value of ρ, as anticipated in the comments about Figure 6.

On the other hand, Figure 7b suggests a much better robustness of the GID, mGID, and PRIDe than the other detectors; an equivalent conclusion regarding the GID has been reported in [13]. Since Figure 7a adopts the correct way of computing the SNR as the average of the quotient between the instantaneous signal and noise powers, the conclusions drawn from it are the correct ones. In Figure 7b, the probability of detection for all detectors are overestimated at higher values of ρ, since the actual (correct) SNRs in this case have been measured as −12.98, −12.93, −12.73, −12.37, −11.61, and −8.74 dB, respectively, for ρ=0, 0.2, 0.4, 0.6, 0.8, and 0.99 (recall that the SNR associated with Figure 7b has been configured as −13 dB for any ρ, applying the incorrect definition). Observe that if −13 dB is subtracted from the above SNR values, the results are approximately the quantities given in Figure 1.

To put it concisely, the detectors GID, mGID, PRIDe, HR, VD1, and GRCR show some resistance to changes in noise power, but their level of robustness is not as high as perceived when an incorrect definition of the SNR is utilized.

**Figure 8:**
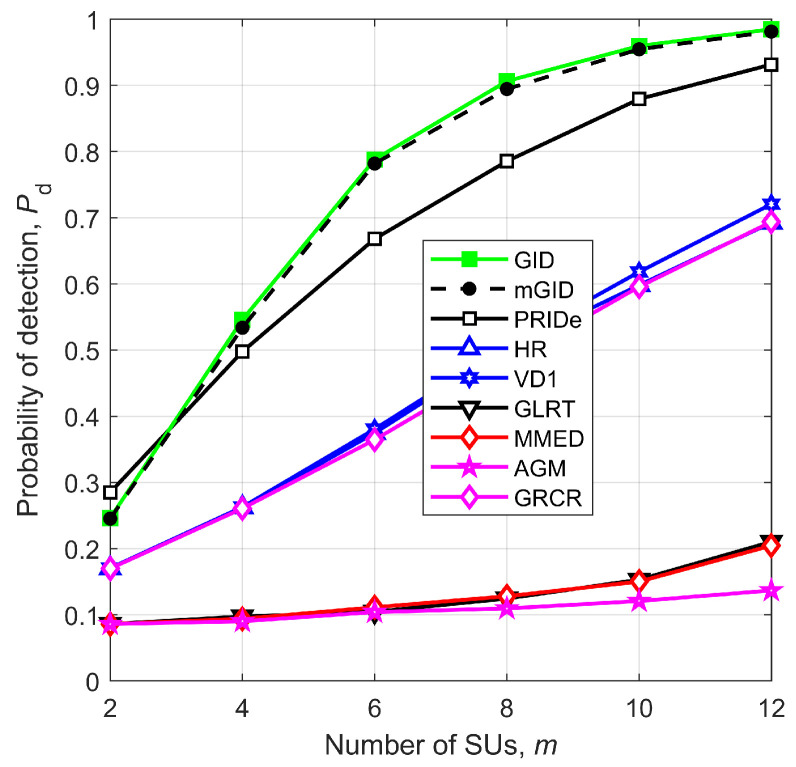
$P_{d}$ versus *m* for SNR=−14 dB, η=2.5, r=1 km, n=250, ρ=0.5, $\mu_{K}=20$ dB, and $\sigma_{K}=0$ dB.

Figure 8 gives $P_{d}$ versus the number of SUs, *m*, for SNR=−14 dB, η=2.5, r=1 km, n=250, ρ=0.5, $\mu_{K}=20$ dB, and $\sigma_{K}=0$ dB. As expected, the performances of all robust detectors improve as *m* increases, whereas the non-robust ones (GLRT, MMED, and AGM) do not follow this improvement pattern due to the deleterious influence of the signal and noise level variations. For the system settings considered in Figure 8, the GID and mGID considerably outperform the other detectors, closely followed by the PRIDe. Once again, the results show practically identical performances of the GID and mGID.

**Figure 9:**
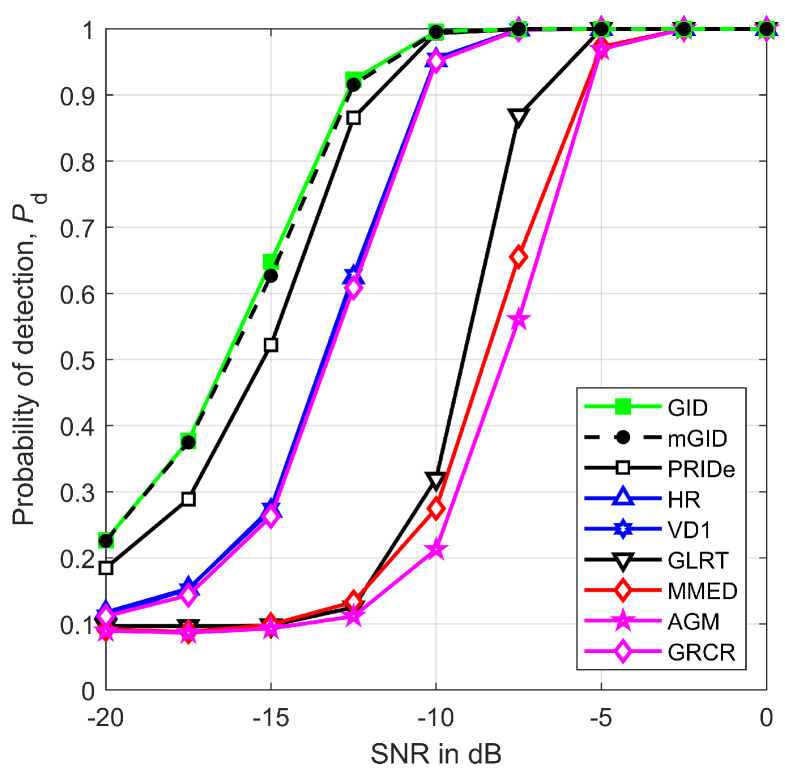
$P_{d}$ versus SNR for m=6, η=2.5, r=1 km, n=250, ρ=0.5, $\mu_{K}=20$ dB, and $\sigma_{K}=0$ dB.

Figure 9 depicts $P_{d}$ versus the SNR, in dB, assuming m=6, η=2.5, r=1 km, n=250, ρ=0.5, $\mu_{K}=20$ dB, and $\sigma_{K}=0$ dB. The performance improvement observed for all detectors as the SNR increases is the expected outcome. The GID and mGID performances, once again, are in agreement with each other, and are superior to the other detectors, given that the line-of-sight condition has been established by setting $\mu_{K}=20$ dB. The PRIDe performs quite similarly to the GID and mGID, while the HR, VD1, and GRCR come in third position with practically identical performances. It can be seen that, in spite of being under nonuniform received signal and noise powers, the GLRT, MMED, and AGM are capable of attaining satisfactory performances, but only at relatively higher SNR regimes.

**Figure 10:**
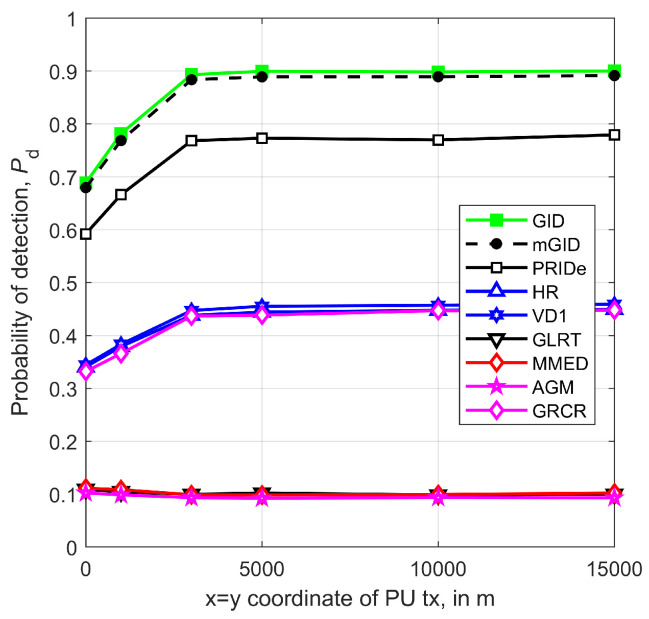
$P_{d}$ versus (x,y) coordinates of the PU transmitter, x=y, for m=6, SNR=−14 dB, η=2.5, r=1 km, n=250, ρ=0.5, $\mu_{K}=20$ dB, and $\sigma_{K}=0$ dB.

The influence, on $P_{d}$, of the distances from the PU to the SUs, which depend on the (x,y) coordinates of the PU, is shown in Figure 10 for x=y, m=6, SNR=−14 dB, η=2.5, r=1 km, n=250, ρ=0.5, $\mu_{K}=20$ dB, and $\sigma_{K}=0$ dB. The non-robust detectors (GLRT, MMED, and AGM) are useless for any PU coordinate, which is owed to the variability in signal and noise levels and to the SNR value (see Figure 9). When the PU coordinates x,y are below ≈3r m, the performances of all robust detectors decrease due to the larger relative discrepancies among the received signal powers, similarly to what happens when the path-loss exponent increases. For x,y above ≈3r m, practically no performance changes are observed for all detectors, meaning that the relative discrepancies among the received signal powers were not capable of causing performance loss or improvement.

**Figure 11:**
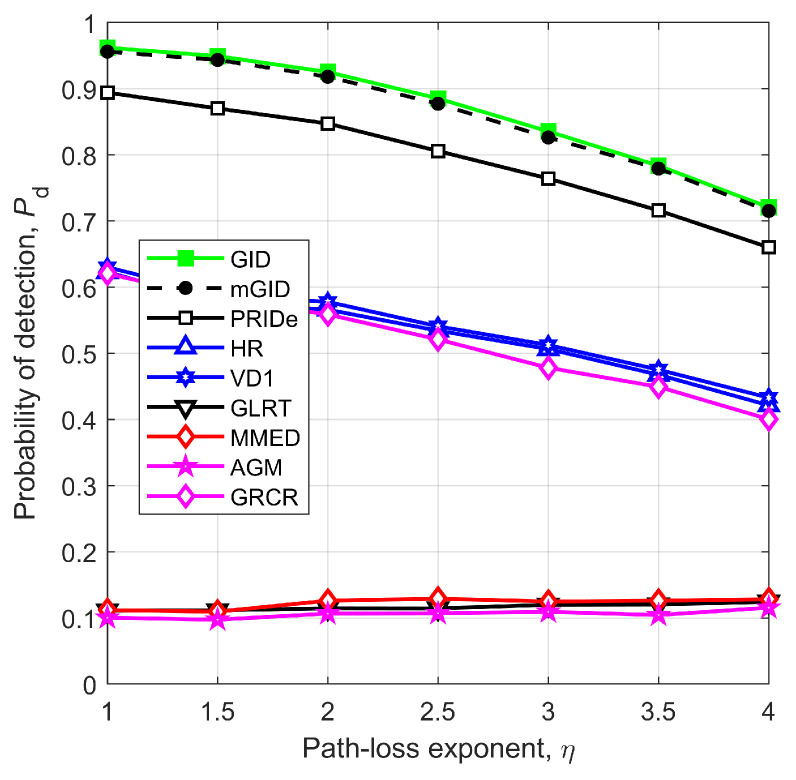
$P_{d}$ versus η for m=6, SNR=−13 dB, r=1 km, n=250, ρ=0.5, $\mu_{K}=20$ dB, and $\sigma_{K}=0$ dB.

Finally, Figure 11 gives $P_{d}$ as a function of the path-loss exponent, η. The detectors GLRT, MMED, and AGM attain useless performances for any η, a consequence of their non-robustness to the noise and signal power variations. The performances of the remaining detectors reduce as η becomes larger, which seems an expected outcome if one recalls that a larger path-loss exponent means higher signal attenuation from the PU to the SUs. However, the performance reduction shown in Figure 11 is not directly caused by the higher attenuation, since the SNR has been set as fixed for any η. A higher path-loss exponent produces higher differences among the received signal power levels, which translate into worse spectrum sensing performances. For instance, a realization of the received signal powers, in dBm, for η=1 was 14.4, 14.0, 13.9, 16.4, 17.9, and 14.5. For η=4, another realization of the signal powers, also in dBm, was −79.7, −75.1, −80.2, −75.2, −65.8, and −65.9. Notice the higher variations in the second case.

## 5. Conclusions and Opportunities for Future Research

The modified GID (mGID) has been proposed in this article. It has been observed that the mGID inherited the attractive attributes of the GID, additionally having a computational cost far below the GID. The time complexity of the mGID obeys approximately the same growth rate of the GID, but has a constant factor approximately 23.4 times smaller. In other words, the mGID spends approximately 4% of the processing time spent to calculate the GID test statistic, which corresponds to a huge reduction in the latency of the spectrum sensing process. Furthermore, this latency reduction has come with no performance loss in the mGID with respect to the GID.

Two improvements to the models originally proposed in [13] have also been suggested: (i) the variations in the received signal levels have been associated with the path loss of the channels between the PU transmitter and the SUs; and (ii) the models for the SNR computation and calibration of the corresponding noise power level have been modified to take into account the correct definition of the SNR as the expectation of the quotient between the random signal and noise power levels. Besides more trustable results, these improvements have uncovered the actual degree of robustness of the analyzed detectors against time-varying signal and noise levels, which is not as high as previously reported in the literature.

A fully-commented Matlab code has been made available at [16], allowing a complete performance analysis of the detectors considered herein under a variety of circumstances and system parameters. Other detectors can be easily added to the analysis by simply adapting or reproducing the sub-routines inside the code.

Some opportunities for future research departing from the present work can be highlighted: (i) the hardware implementation of the mGID in a field-programmable gate array (FPGA), an application-specific integrated circuit (ASIC), or both, is a natural path beyond the contributions of this article; (ii) the model described in Section 2 is already an appealing and practical model, but could be improved by adding spatially correlated shadowing and erroneous report channels to allow for the assessment of their influences on the spectrum sensing performance; (iii) in a simpler work, other detectors could be also compared with the mGID in terms of performance and computational cost.

## Abbreviations

The following abbreviations are used in this manuscript:

AGM	Arithmetic to geometric mean
ASIC	Application-specific integrated circuit
AWGN	Additive white Gaussian noise
CDF	Cumulative distribution function
CR	Cognitive radio
CSS	Cooperative spectrum sensing
DSA	Dynamic spectrum access
FC	Fusion center
FPGA	Field programmable gate array
GID	Gini index detector
GLRT	Generalized likelihood ratio test
GRCR	Gerschgorin radii and centers ratio
HR	Hadamard ratio
IoT	Internet of Things
LNA	Low-noise amplifier
LoS	Line of sight
mGID	Modified Gini index detector
MMED	Maximum-minimum eigenvalue detector
mPRIDe	Modified Pietra–Ricci index detector
PDF	Probability density function
PRIDe	Pietra-Ricci index detector
PU	Primary user
QPSK	Quadrature phase-shift keying
RF	Radio frequency
SCM	Sample covariance matrix
SNR	Signal-to-noise ratio
SU	Secondary user
VD	Volume-based detector
VD1	Volume-based detector number 1
VD2	Volume-based detector number 2
5G	Fifth generation
6G	Sixth generation
